# Circulating level of microRNA-142-5p is a potential biomarker for predicting in-stent restenosis: a case–control study

**DOI:** 10.1186/s12872-021-01893-y

**Published:** 2021-02-08

**Authors:** Chun-Hsu Pan, Shu-Chen Chien, Chang-Jui Chen, Chun-Ming Shih, Ming-Hsiung Hsieh, Chun-Yao Huang, Wei-Fung Bi, Chao-Shun Chan, Yung-Ta Kao, Cheng-Yi Hsiao, Shuo-Ju Chiang, Kuang-Hsing Chiang, Jen-Hung Huang, Yun-Ru Liu, Ji-Dung Luo, Hui-Yu Huang, Chieh-Hsi Wu

**Affiliations:** 1 PhD Program in Drug Discovery and Development Industry, College of Pharmacy, Taipei Medical University, Taipei, 11031 Taiwan; 2 School of Pharmacy, Taipei Medical University, Taipei, 11031 Taiwan; 3 Department of Pharmacy, Taipei Medical University Hospital, Taipei, 11031 Taiwan; 4 Department of Cardiology, Taipei Medical University Hospital, Taipei, 11031 Taiwan; 5 Department of Cardiology, Taipei Municipal Wanfang Hospital, Taipei Medical University, Taipei, 11031 Taiwan; 6 Joint Biobank, Office of Human Research, Taipei Medical University, Taipei, 11031 Taiwan; 7 Bioinformatics Resource Center, The Rockefeller University, New York, NY 10065 USA; 8 Graduate Institute of Metabolism and Obesity Sciences, Taipei Medical University, Taipei, 11031 Taiwan

**Keywords:** Biomarker, Circulating level, In-stent restenosis, miRNA

## Abstract

**Background:**

Patients who receive percutaneous coronary intervention (PCI) have different chances of developing in-stent restenosis (ISR). To date, no predictable biomarker can be applied in the clinic. MicroRNAs (miRNAs or miRs) play critical roles in transcription regulation, and their circulating levels were reported to have potential as clinical biomarkers.

**Methods:**

In total, 93 coronary stent-implanted patients without pregnancy, liver or renal dysfunction, malignancy, hemophilia, or autoimmune diseases were recruited in this clinical study. All recruited participants were divided into an ISR group (*n* = 45) and a non-ISR group (*n* = 48) based on their restenotic status as confirmed by cardiologists at the first follow-up visit (6 months after surgery). Blood samples of all participants were harvested to measure circulating levels of miRNA candidates (miR-132, miR-142-5p, miR-15b, miR-24-2, and miR-424) to evaluate whether these circulating miRNAs can be applied as predictive biomarkers of ISR.

**Results:**

Our data indicated that circulating levels of miR-142-5p were significantly higher in the ISR population, and results from the receiver operating characteristic (ROC) curve analysis also demonstrated superior discriminatory ability of miR-142-5p in predicting patients’ restenotic status. In addition, circulating levels of miR-15b, miR-24-2, and miR-424 had differential expressions in participants with diabetes, hyperlipidemia, and hypertension, respectively.

**Conclusions:**

The current study revealed that the circulating level of miR-142-5p has potential application as a clinical biomarker for predicting the development of ISR in stent-implanted patients.

## Background

Restenosis after a percutaneous coronary intervention (PCI) has historically been described as an “Achilles heel” [1]. Although the invention of drug-eluting stents (DESs) improved the restenosis incidence rate to 10–15%, compared to the 25–30% rate of bare-metal stents (BMSs), restenosis remains a clinical challenge in the real world [2]. Despite a higher restenosis rate with BMSs than with DESs, the much-higher cost of DESs and uncertain safety and efficacy are factors favoring BMS use [3]. Numerous studies have investigated the question of whether DESs outperform BMSs. Piccolo and colleagues recently conducted a meta-analysis of 20 randomized control trials to compare the safety and efficacy performance between new-generation DESs and BMSs [4]. This elicited the question as to whether the BMS option is still needed in current practice. Colombo and colleagues addressed this issue and remarked that BMS management should remain in current practice, especially for patients who cannot complete dual antiplatelet therapy, patients with high-bleeding risks, and patients who need non-cardiac surgery [5]. Therefore, the best strategy is to categorize patients and thus optimize personalized management when choosing a stent.

MicroRNA (miRNAs or miRs) are small noncoding RNAs (with ca. 21–25 nucleotides) that play vital roles in post-transcription of specific messenger (m)RNA targets [6]. Studies demonstrated that miRNAs regulate nearly all physiological processes and are associated with several human diseases, including cancer, angiogenesis, immune-related diseases, and neurodegenerative diseases [7–10]. In fact, miRNAs are recognized as disease indicators, because expressions of distinct miRNAs may indicate specific cancer types. Also, miRNAs with characteristic roles reflect different hematopoietic lineages [11]. In addition, noninvasive and sensitive features of miRNAs have attracted attention due to their potential as a tactic in tackling this “Achilles heel” challenge [12]. As a result, miRNAs could be the best fit to investigate the complex restenosis pathology with their disease-specific and ideal-biomarker characteristics. Several miRNAs, including miR-132, miR-142-5p, miR-15b, miR-24-2, and miR-424, were reported to modulate cellular behaviors or functions in vascular smooth muscle cells and endothelial cells as well as to regulate restenotic progression in animal studies [13–21]. The objective of this study was to clarify correlations of circulating levels of five miRNAs and the prevalence of in-stent restenosis (ISR) in patients who had received vascular stent implantation.

## Methods

### Clinical study

This study was approved by the Taipei Medical University Joint Institutional Review Board (TMU-JIRB No. 201405019), and all experiment protocols were in accordance with the Declaration of Helsinki. All participants signed informed consent forms and were recruited by cardiologists at Taipei Medical University Hospital and Taipei Municipal Wanfang Hospital from June 2015 to May 2016. The inclusion criterion was patients who had undergone PCI, and exclusion criteria were patients who were pregnant, and those who had been diagnosed with liver diseases (liver dysfunction), kidney dysfunction (dialysis), malignancy, hemophilia, or autoimmune diseases. In total, 93 patients were recruited and analyzed. Participants were divided into two groups (ISR and non-ISR) based on their restenotic status as confirmed by cardiologists at the first follow-up visit (6 months after surgery). The ISR group was defined as having a greater than 50% decrease in the luminal diameter. Patients were enrolled by cardiologists and needed to have 15 mL of blood taken for analysis. Blood was obtained by a certificated medical technologist or nurse in each medical facility. Blood was collected in anticoagulant tubes and was stored at 2–8 °C until further processing.

### Purification and reverse transcription (RT) of miRNA

Serum miRNA was purified using a miRNeasy Serum/Plasma Mini Kit (#217004, Qiagen, Venlo, the Netherlands) according to the original manufacturer’s protocol. The RT reaction of miRNA for each sample was performed using a miScript II RT kit (#218161, Qiagen) to obtain complementary (c)DNA based on the manufacturer’s instructions.

### Quantitative polymerase chain reaction (qPCR)

Serum miRNA expression was measured in plasma of each patient using a qPCR. U6 non-coding small nuclear (U6 sn)RNA was employed as an interval control. Primers were synthesized by Qiagen, and primer sequences are presented in Table 1. The miScript SYBR Green PCR kit (#218073, Qiagen) and miScript Primer Assay (#218300, Qiagen) were used with the CFX96™ Real-Time PCR Detection System (Bio-Rad, Hercules, CA, USA) to enable quantification of mature miRNA. Cycling conditions for the PCR were as follows: initial activation at 95 °C for 15 min, followed by 40 cycles of three-step cycling including denaturation at 94 °C for 15 s, annealing at 55 °C for 30 s, and extension at 70 °C for 30 s, at which time, fluorescence data were collected. This procedure was repeated three times for each sample with a total reaction volume of 25 μL. The relative transcript expression was calculated using the equation. 2^–ΔΔCt^.

**Table 1:** Sequences of primers used for detecting micro (mi)RNAs

Target gene	miRBase accession	Primer sequences
miR-132	MIMAT0000426	UAACAGUCUACAGCCAUGGUCG
miR-142-5p	MIMAT0000433	CAUAAAGUAGAAAGCACUACU
miR-15b	MIMAT0000417	UAGCAGCACAUCAUGGUUUACA
miR-24-2	MIMAT0004497	UGCCUACUGAGCUGAAACACAG
miR-424	MIMAT0001341	CAGCAGCAAUUCAUGUUUUGAA
U6 snRNA	–	CTCGCTTCGGCAGCACA AACGCTTCACGAATTTGCGT

^*^The miScript Universal Primer (Qiagen) was used as a reverse primer for detecting mRNAs

### Statistical analysis

All values are presented as the mean ± standard deviation (SD). Differences among multiple groups were determined using a one-way analysis of variance (ANOVA) in combination with Dunnett's test. A value of *p* < 0.05 was considered statistically significant. Statistical assays were performed using SAS software (vers. 9.4; SAS Institute, Cary, NC, USA). A receiver operating characteristic (ROC) curve was drawn to assess the predictive accuracy of the biomarkers. We evaluated the discriminative power of the biomarker candidates by calculating the area under the ROC curve (AUC). A diagnostic test with an AUC of 1.0 was defined as a perfect diagnostic test, whereas an AUC of 0.5 was regarded as a non-discriminating test. Chi-squared test was used to compare correlations of different variables between the restenosis and non-restenosis groups. A logistic regression analysis was performed by a previously described method [22].

## Results

### Patient characteristics

Basic demographic data of recruited patients are shown in Table 2. The recruiting age range was 30–80 years. In the present study, 93 patients were analyzed (including 45 patients in the ISR group and 48 patients in the non-ISR group). Age, clinical comorbidities, and medications were comparable between the ISR and non-ISR groups. Apart from clopidogrel, which significantly differed, there were no other significant differences among clinical characteristics between the ISR and non-ISR groups.

**Table 2:** Clinical demographics of the ISR and non-ISR groups in this study

Parameter	Total (*N* = 93)	ISR (*N* = 45)	Non-ISR (*N* = 48)	*p* value
*Age (years)*	65.71 ± 8.73	65.69 ± 8.82	65.61 ± 8.72	0.12
*Gender*				
Male	76 (81.72%)	41 (91.11%)	35 (72.92%)	–
Female	17 (18.28%)	4 (8.89%)	13 (27.08%)	–
*Smoking*	3 (3.23%)	2 (4.44%)	1 (2.08%)	–
*Comorbidities*				
Hyperlipidemia	84 (90.32%)	42 (93.33%)	42 (87.50%)	0.34
Hypertension	71 (76.34%)	33 (73.33%)	38 (79.17%)	0.51
Angina pectoris	42 (45.16%)	22 (48.89%)	20 (41.67%)	–
Diabetes	29 (31.18%)	15 (33.33%)	14 (29.17%)	0.67
Chronic CHF	5 (5.38%)	3 (6.67%)	2 (4.17%)	–
*Medications*				
Aspirin	75 (80.65%)	40 (88.89%)	35 (72.92%)	0.05
Beta blocker	51 (54.84%)	29 (64.44%)	22 (45.83%)	0.07
CCBs	30 (32.26%)	14 (31.11%)	16 (33.33%)	0.82
ARB	61 (65.59%)	30 (66.67%)	31 (64.58%)	0.83
Clopidogrel	43 (46.24%)	15 (33.33%)	28 (58.33%)	0.02
Lipid-lowering agent	84 (90.32%)	41 (91.11%)	43 (89.58%)	0.80

Data are presented as the mean ± standard deviation or number (percentage). CHF, cardiac heart failure; CCBs, calcium channel blockers; ARB, angiotensin II receptor blocker

### Circulating levels of miRNA candidates

**Fig. 1:**
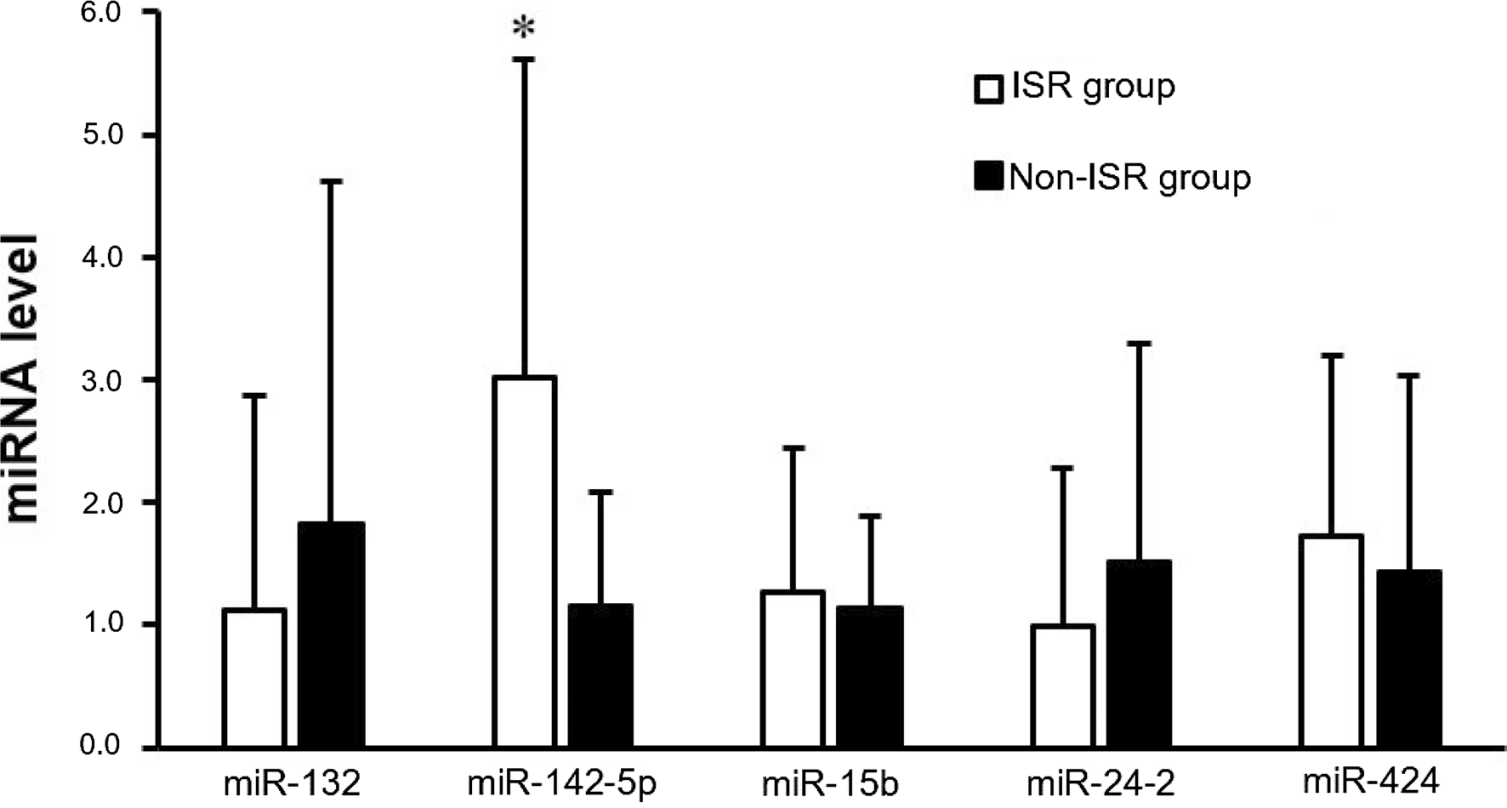
Circulating levels of miRNAs in the ISR and non-ISR populations. * *p* < 0.05, indicates a significant difference between the two groups

To compare the difference in expressions of each miRNA, we performed a qPCR to measure relative levels of circulating miRNAs of the ISR group compared to the non-ISR group. We observed that two miRNAs (miR-132 and miR-24-2) were lower, while the other miRNAs (miR-15b, miR-424, and miR-142-5p) were higher in the ISR population. Previous studies proposed that miR-132 and miR-24-2 might have a protective function against restenotic processes [13, 14, 17, 18]. On the other hand, miR-142-5p showed a significantly higher abundance in the restenosis population over the non-restenosis population. This observation of miR-142-5p expression levels is consistent with our hypothesis, according to a literature review. That is, miR-142-5p may increase the restenosis risk. Interestingly, miR-15b and miR-424 appeared to be slightly higher in the restenosis population compared to the non-restenosis group. Previous studies regarding miR-15b and miR-424 demonstrated their protective and preventive potentials against atherosclerosis development in a murine model, but opposite effects were shown in this human population (Fig. 1).

### ROC performances of miRNA biomarkers and comparisons in human plasma

To test these miRNA roles as clinical prognostic biomarkers, we conducted a ROC analysis and assessed the diagnostic value of each of the five selected miRNAs. Detailed information from the ROC analysis as to the ability of the five miRNAs to diagnose restenosis in patients is shown in Table 3. Consistent with expression level results, miR-142-5p showed the highest predictive value (with an AUC of 0.734), which indicated good diagnostic accuracy [23]. The AUC of miR-424 (0.599) was second behind miR-142-5p, and was on the edge of exhibiting sufficient diagnostic accuracy. The AUC results for miR-15b, miR-132, and miR-24-2 fell in the range considered to have no diagnostic value despite their high specificity for restenosis (at 0.466, 0.425, and 0.364 respectively, with an AUC of < 0.5 considered to have no diagnostic value). These results demonstrated that miR-142-5p had a superior diagnostic performance among the five miRNAs for discriminating patients with from those without a high risk of restenosis.

**Table 3:** Receiver operating characteristic (ROC) curve analysis of miRNAs between the ISR and non-ISR populations

miRNA	AUC	95% CI	*p*	Sensitivity	Specificity	Accuracy
miR-132	0.425	[− 0.209, 0.059]	0.274	0.059	0.973	0.535
miR-142-5p	0.734	[0.093, 0.375]	0.001	0.432	1.000	0.571
miR-15b	0.466	[− 0.061, 0.094]	0.606	0.086	0.821	0.543
miR-24-2	0.364	[− 0.268, − 0.003]	0.045	0.059	0.943	0.507
miR-424	0.599	[− 0.027, 0.226]	0.124	0.692	0.575	0.633

AUC, are under the ROC curve; CI, confidence interval

### Effects of comorbidities in circulating levels of miRNA candidates of participants

Hyperlipidemia, hypertension, angina pectoris, and diabetes are common clinical comorbidities in patients treated with percutaneous transluminal coronary angioplasty (PTCA). It was unclear whether these comorbidities might influence circulating levels of our examined miRNA candidates between the ISR and non-ISR groups. Experimental results showed that circulating miR-142-5p and miR-24-2 significantly differed between the ISR and non-ISR groups in participants with hyperlipidemia (Table 4). Similarly, markedly higher miR-142-5p and miR-424 levels were found in the ISR group in participants with hypertension (Table 5). In participants with diabetes, plasma levels of miR-132 and miR-424 were significantly lower in the ISR group compared to those in the non-ISR group (Table 6). However, angina pectoris had no impact on circulating levels of any of these miRNA candidates between the ISR and non-ISR groups (Table 7).

**Table 4:** Comparison of miRNA levels between the ISR and non-ISR groups in participants with hyperlipidemia (*N* = 84)

miRNA	Group		
	ISR	Non-ISR	*p* value
miR-132	1.055 ± 1.750	1.963 ± 2.908	0.074
miR-142-5p	3.047 ± 2.521	1.186 ± 0.971	0.019^a^
miR-15b	1.217 ± 1.153	1.086 ± 0.676	0.519
miR-24–2	0.835 ± 0.934	1.607 ± 1.884	0.022^a^
miR-424	1.664 ± 1.531	1.544 ± 1.646	0.450

^a^Indicates a statistical difference between the two groups

Data are presented as the mean ± standard deviation

**Table 5:** Comparison of miRNA levels between the ISR and non-ISR groups in participants with hypertension (*N* = 71)

miRNA	Group		
	ISR	Non-ISR	*p* value
miR-132	1.313 ± 2.005	1.629 ± 2.439	0.587
miR-142-5p	2.817 ± 1.928	1.297 ± 1.010	0.026^a^
miR-15b	1.284 ± 1.195	1.111 ± 0.693	0.956
miR-24-2	0.919 ± 0.990	1.528 ± 1.967	0.281
miR-424	2.058 ± 1.620	1.287 ± 1.066	0.028^a^

^a^Indicates a statistically significant difference between the two groups

Data are presented as the mean ± standard deviation

**Table 6:** Comparison of miRNA levels between the ISR and non-ISR groups in participants with diabetes (*N* = 29)

miRNA	Group		
	ISR	Non-ISR	*p* value
miR-132	1.530 ± 2.276	3.664 ± 4.368	0.047^a^
miR-142-5p	3.307 ± 2.578	1.233 ± 1.358	0.097
miR-15b	1.213 ± 1.137	1.767 ± 0.971	0.033^a^
miR-24–2	1.613 ± 2.023	2.558 ± 2.805	0.412
miR-424	1.778 ± 1.594	2.007 ± 2.432	0.801

^a^Indicates a statistically significant difference between the two groups

Data are presented as the mean ± standard deviation

**Table 7:** Comparison of miRNA levels between the ISR and non-ISR groups in participants with angina pectoris (*N* = 31)

miRNA	Group		
	ISR	Non-ISR	*p* value
miR-132	0.563 ± 0.394	1.221 ± 1.350	0.277
miR-142-5p	2.195 ± 1.409	1.496 ± 1.368	0.637
miR-15b	0.797 ± 0.529	0.819 ± 0.314	0.563
miR-24–2	0.498 ± 0.314	1.118 ± 0.763	0.060
miR-424	1.590 ± 1.264	1.227 ± 0.57	0.392

Data are presented as the mean ± standard deviation

## Discussion

ISR refers to a reduction in coronary vessel diameter after a PCI, which remains a major clinical problem in the cardiovascular field. The pathological processes of restenosis are known to be complicated due to the many factors involved [24]. Some biological and genetic factors, such as drug resistance [25], systemic and allergic inflammation [26, 27], neoatherosclerosis [28], variants of the β2-adrenergic receptor [29], and polymorphisms of the tumor necrosis factor-α gene [30], were reported to participate in the progression of DES restenosis. Subclinical hypothyroidism and inflammatory activity were also demonstrated to regulate the stability of atherosclerotic plaques isolated from internal carotid artery stenosis [31]. Diabetes is a risk factor which strikingly increases the prevalence of, incidence of, and mortality due to cardiovascular diseases [32]. ISR was confirmed to be positively associated with mean blood glucose levels, and strict peri-procedural glycemic control could improve outcomes after a PCI in patients with an acute ST-elevation myocardial infarction [33]. Pathophysiological activation of the ubiquitin proteasome system was shown to be involved in several pathological characteristics (e.g., endothelial dysfunction and plaque destabilization) during atherosclerosis progression in diabetic patients and to be one of the mechanisms causing the generation of insulin resistance [34]. Moreover, the prospective AIRE Study indicated that lower levels of serum adiponectin, an adipocyte-derived factor with an antidiabetic effect, were related to restenosis even in subjects with normal glucose tolerance [35]. Epigenetic markers are reversible, which can reprogram genetic dysregulation independent of inherited DNA sequences. Therefore, epigenetic merits are applied to prognostic biomarkers and even therapeutic targets in various fields. miRNAs are one kind of emerging epigenetic marker in advancing cardiovascular research during the last decade [36, 37]. However, molecular mechanisms linking epigenetic and genetic phenomena are still lacking. Moreover, miRNA biomarker research in restenosis remains limited. The present study attempted to investigate potential miRNA biomarkers to characterize restenosis risk levels among patients who had undergone PCI management by analyzing genetics to further explore molecular mechanisms.

Although several miRNAs have been studied and found to be involved in angiogenesis or vascular neointima, our findings in human clinical samples highlight crucial aspects that have, to our knowledge, not been underscored before. First, Xu et al. argued that miR-15b modulates the smooth muscle cell phenotype through targeting the yes-associated protein (YAP) in a rat model, which may decrease the restenosis risk [38]. Our data demonstrated an opposite phenomenon when translated from a rat model to human samples. Costa de Freitas et al. proved that clopidogrel exposure modulates miR-15b-5p and miR-26a-5p expression levels in vitro [39]. In our study, 46.24% of participants were using clopidogrel, and the data showed a significant difference between the restenosis and non-restenosis populations. As opposed to the animal model, clinical human-environmental influences are much more complicated. Medication administration serves an essential role in regulating epigenetic marker performance. Second, Muñoz-Pacheco et al. indicated that ezetimibe suppresses the extracellular signal-regulated kinase (ERK)/mitogen-activated protein kinase (MAPK) pathway and nuclear factor (NF)-κB activity and thus downregulates miR-424 expression. Those findings suggest that ezetimibe’s anti-atherosclerotic effects occur through inhibiting miRNAs [40]. Around 90% of our participants were using lipid-lowering agents. That may explain the discrepancy in the hypothetical role of miR-424 in restenosis prognosis. Interestingly, Richardsen et al. recently argued that low expression of miR-424 was correlated with an aggressive prostate cancer phenotype, and thus may worsen clinical outcomes and failure. Richardsen et al. concluded that the roles of miR-424 in regulating host immune responses among different cancer types remain to be further elucidated [41]. Not only medical use, but also the disease state and type are also critical points determining miRNA epigenetic marker regulation. Last, miRNAs have been characterized as ideal biomarkers due to their specific, sensitive, predictive, and non-invasive features [42, 43]. The ROC analysis of these five miRNAs presented their potential performance in stratifying risk levels for restenosis.

Some limitations of this study might be of concern. First, the sample size of recruited patients was small, and it might not have sufficient power to fully clarify relationships between circulating levels of miRNA candidates and the restenotic status under different conditions (e.g., comorbidities, age, gender, medicinal interventions, diet, or lifestyle). Second, this project was not a clinical study that recruited multiple racial participants; thus these data might not be applicable to different ethnic groups. Finally, long-term follow-up with multiple observation times are needed to clarify whether correlations between circulating miRNAs and the restenotic status change over time.

## Conclusions

Personalized medicine has potential benefits to substantially improve outcomes of clinical interventions by the tailored therapies based on individual patient’s characteristics, such as genetic information, physiological status, diet and lifestyle. Predictable biomarkers of ISR could be clinically applied for reducing wasteful practices by helping us to distinguish precisely who belongs to the susceptible population and then the most suitable intervention (e.g., bare-metal stent or drug-eluting stent implantation) could be chosen for individual patients. In this study, we established an analysis of dynamic molecular mechanisms through epigenetic human clinical trials of complicated restenosis pathological processes. The data indicated that miR-142-5p has significant potential to predict the population at high risk of restenosis, while miR-132 and miR-24-2 may be beneficial in decreasing the restenosis risk. These miRNA epigenetic markers demonstrated the real-time and dynamic state of individuals with their comorbidities and medication use. Our study offers potential biomarker tools and possible molecular mechanisms for targeting in-stent restenosis.

## Data Availability

The data used to support the findings of this study are available from the corresponding author upon reasonable request.
